# Creative Music Therapy with Premature Infants and Their Parents: A Mixed-Method Pilot Study on Parents’ Anxiety, Stress and Depressive Symptoms and Parent–Infant Attachment

**DOI:** 10.3390/ijerph18010265

**Published:** 2020-12-31

**Authors:** Selina M. Kehl, Pearl La Marca-Ghaemmaghami, Marina Haller, Elisabeth Pichler-Stachl, Hans Ulrich Bucher, Dirk Bassler, Friederike B. Haslbeck

**Affiliations:** 1 Department of Neonatology, University Hospital Zurich, University of Zurich, Frauenklinikstrasse 10, 8091 Zurich, Switzerland; selina.kehl@kispi.uzh.ch (S.M.K.); buh@usz.ch (H.U.B.); Dirk.Bassler@usz.ch (D.B.); 2 Department of Clinical Music Therapy, Zurich University of the Arts, Pfingstweidstrasse 96, 8005 Zurich, Switzerland; 3 Department of Neonatology and Pediatric Intensive Care, Children’s University Hospital of Zurich, Steinwiesstrasse 75, 8032 Zurich, Switzerland; 4 Department of Clinical Psychology and Psychotherapy, University of Zurich, Binzmuehlestrasse 14/Box 26, 8050 Zurich, Switzerland; pearl.lamarca@peace-academy-society.org; 5 Psychology Research and Counselling Institute for Sexuality, Marriage and Family, International Academy for Human Sciences and Culture, Staadweg 3, P.O. Box 57, 8880 Walenstadt, Switzerland; 6 Department of Psychological Methods, Evaluation and Statistics, University of Zurich, Binzmuehlestrasse 14/Box 27, 8050 Zurich, Switzerland; marina.haller@psychologie.uzh.ch; 7 Division of Neonatology, Department of Pediatrics, Medical University, Auenbruggerplatz/38/III. OG, 8036 Graz, Austria; Elisabeth.Pichler-Stachl@klinikum-graz.at

**Keywords:** music therapy, NICU, prematurity, postnatal anxiety, postnatal stress, attachment

## Abstract

Premature birth is stressful for infants and parents and can adversely affect the parent–infant dyad. This mixed-methods pilot study evaluates whether creative music therapy (CMT) can alleviate anxiety, stress, and depressive symptoms in parents and support the bonding process with their infant. Sixteen parent couples were included. Ten couples were randomly allocated to the music therapy group (MTG) and six to the control group (CG). All couples completed psychological questionnaires measuring anxiety and depressive symptoms as well as an implicit measure of parent–infant attachment at two weeks postpartum (T1), at approximate neonatal intensive care unit (NICU) hospitalization halftime (T2), and two weeks after the infant had been discharged (T3). At T1 and T2, the parents additionally completed a questionnaire assessing the degree of stress they experienced at the NICU. Qualitative data were collected through a semi-structured, problem-centered interview with MTG parents at T3. The results of the quantitative analyses revealed reductions in anxiety levels from T1 to T2 (*p* = 0.002) as well as decreases in depressive symptoms from T2 to T3 (*p* = 0.022). No such changes were apparent in the CG. In fact, parental stress increased from T1 to T2 (*p* = 0.016). Significant increases in attachment across time were also observed within the MTG, but not in the CG. The qualitative inquiry confirmed that CMT can support the parent–infant relationship. Being in musical interaction evoked feelings of joy and relaxation in the parents and encouraged them to interact more profoundly with their infant. The results call for a more extensive powered follow-up study to further investigate CMT’s potential for parental well-being and parent–infant bonding.

## 1. Introduction

Premature birth is a major challenge for both premature infants and their parents. Due to the perinatal health care system’s improvement, many more premature infants survive, but undesirable long-term effects of preterm birth range from deficits in executive functions to socio-emotional and behavioral problems [1,2].

Parents of premature infants bear the burden of this situation as well. In addition to the shock of premature birth, they face numerous further challenges and difficulties: the early separation from their child is traumatizing, and not being able to fully care for their infant leads to a lack of autonomy as primary caregivers [3,4]. These two factors are experienced as major stressful events by parents of premature infants [4]. Added to this, the neonatal intensive care unit (NICU) remains a stressful environment where privacy and quiet are rare. Instead, bright lights, beeping monitors and odors are predominant [5]. Moreover, premature infants have to undergo various repeated and cumulative pain procedures [6]. Yet, the implementation of pain management strategies, which include pharmacological (e.g., opioid administration) and nonpharmacological approaches (e.g., breastfeeding) is currently viewed as inadequate and in need of optimization [7,8,9]. Consequently, and understandably, parents of premature infants cared for in the NICU worry about their infant’s suffering, and experience feelings of helplessness, guilt, and stress [5,10]. Other emotional reactions of which parents of premature infants may suffer, due to the experience of premature birth, include feelings of disappointment, fear, sadness, depression, anger, grief, and loss of self-esteem [5,11].

Consequently, premature infants’ parents may show increased stress and anxiety symptoms and a higher risk of depression than parents of term infants [12,13,14]. Symptoms of traumatization can occur up to several weeks after hospitalization and, if unresolved, may lead to posttraumatic stress disorder [3].

Besides, premature infants have difficulties communicating their internal states [15]. Due to their immaturity, their non-verbal expression is more complex to read than that of a child born at term, making it more difficult for the parents to understand and communicate appropriately with their premature infant [16].

Taken together, these problems may impact upon the parent–infant bonding process. Recent studies indicate that premature birth influences the dyadic quality of the mother–infant interaction [16,17,18], and possibly affects later attachment behavior as well [19]. This at a time when bonding is of considerable importance and especially vital for the development of a child [2,16,20].

Therapeutic intervention programs play an important role in detecting and counteracting possible stress symptoms in mothers of premature infants and supporting the mother–infant relationship [3,21,22].

Creative music therapy (CMT) is an individualized and family-integrating music therapy method [23,24,25]. Based on the concepts of Nordoff–Robbins’ creative music therapy [26] and combined with principles of CMT used on comatose patients [27], it addresses the needs and resources of premature infants and their parents in the NICU setting [25].

During CMT, the breathing pattern, gestures, and facial expressions of the premature infant are perceived by the therapist and transformed into a musical response by humming in a lullaby style [25,28]. The parents are individually involved in the therapeutic process, e.g., by being supported to use their voice in an infant-directed and responsive way or by performing CMT during kangaroo care [24].

A recent pilot randomized control trial (RCT) provided evidence for CMT’s beneficial effects on functional brain activity and brain connectivity in premature infants [29]. However, CMT not only ensures physical progress, stabilization and relaxation, it also addresses and awakens the communicative abilities of premature infants, which has been demonstrated in qualitative case studies [25,28].

Finally, CMT aims to address the parents’ needs and to promote the parent–infant bonding process. By intuitively relating through music, parents are given the opportunity to connect and care for their infant, and the infant’s needs for intimacy and closeness are met [24].

A meta-analysis conducted by Bieleninik et al. [30] confirmed various beneficial music therapy effects for premature infants and their parents. In particular, evidence was found for the positive impact of music therapy on premature infants’ respiratory rate and maternal anxiety.

Nevertheless, to date, only a few RCT studies have examined whether music therapy may alleviate symptoms of anxiety, depression and stress in parents of premature infants [31,32,33]. Moreover, no study so far has researched the effect of CMT on these symptoms. The attachment between parents and their premature infant using a live music therapy approach and its effect over a longer period of time has likewise been rarely evaluated [31].

To address this gap, we aimed to determine how CMT affects symptoms of anxiety, depression, stress, as well as parent–infant attachment of parents of premature infants throughout the duration of their infant’s hospitalization.

Based on previous research findings in the field of music therapy and parents of premature infants [31], we hypothesize that parents of premature infants treated with CMT experience less anxiety and depressive symptoms than parents without CMT (directional hypothesis). We also tested the non-directional hypothesis that CMT would lead to a difference in parents of premature infants compared to parents without CMT in terms of stress experience and parent–infant attachment.

## 2. Materials and Methods

### 2.1. Overview

We chose a mixed-method approach for this study which is part of a larger pilot RCT on the effects of creative music therapy (CMT) on brain function and brain structure in premature infants [29,34]. This sub-study addresses the effect of CMT on parental symptoms of anxiety, depression, stress as well as parent–infant attachment.

The larger study was designed as a prospective, single-center, between-subject randomized, clinical pilot trial in preparation for a full-scale trial and is registered at ClinicalTrials.gov (Number: NCT02434224). Ethical approval was obtained from the Ethics Committee of the Canton of Zurich, Switzerland (KEK-ZH 2014-0655) and the Swiss Agency for Therapeutic Products (Swissmedic), Audit No.: CTCQA14. All parents provided written informed consent before study participation [29,34].

This sub-study was approved by the Ethics Committee of the Canton of Zurich, Switzerland, as an amendment to the broader study (BASEC-Nr. PB_2016-02589). Its sample size is smaller as the sub-study started 26 months later than the main pilot RCT.

### 2.2. Participants

The participants were mothers and fathers of prematurely born infants recruited after birth at the Department of Neonatology of the University Hospital Zurich, Switzerland.

Parents of clinically stable infants born before 32 weeks of gestation and at a chronological age of ≥7 days of life were eligible for study participation. Exclusion criteria were parents of premature infants with a genetically defined syndrome, severe congenital malformation adversely affecting life expectancy and/or neurodevelopment, high-grade intraventricular hemorrhage, and/or cystic white matter lesions. Parents of premature infants admitted to palliative care were also excluded from the study. Lack of sufficient knowledge of the German language was a further defined exclusion criterion.

Infants and parents were randomly allocated to either the music therapy group (MTG) or the control group (CG). The randomization process was managed by the Clinical Trials Center (CTC) of the University Hospital Zurich and its details have been described previously [29]. Preterm infants and their parents allocated to the CG received standard care from the hospital team and skin-to-skin care with their parents. Preterm infants and their parents allocated to the MTG additionally received CMT from one well-trained and highly experienced music therapist (F.B.H.) for at least 8 sessions. As this study is a sub-study of the main study, no sample size calculation was performed.

### 2.3. Intervention

CMT was offered 2–3 times per week in the morning after feeding time following the clinical practice protocol of CMT [23] as well as the study protocol [34].

Each intervention lasted about 20 min and took place, whenever possible, in the presence of the parents (or one parent) who were in skin-to-skin contact with their premature infant. If the parents could not be present (due to a conflicting schedule, such as attending a meeting with the pediatricians and nurses or caring for a sibling at home), the therapeutic session was conducted at the bedside with the premature infant alone, using initial and therapeutic touch as well as the music therapist’s voice. After a period of observation, the music therapist started to hum or sing according to the breathing rhythm, facial expression, and gesture of the premature infant [25,28].

Whenever the parents could participate in the therapeutic sessions, a vibro-acoustic monochord was used to accompany the singing. It was placed on one parent’s elbow so that the vibrations could be felt. A monochord is a wooden, single-stringed instrument manufactured for therapeutic purposes. When touched, its vibro-acoustic stimulation can serve to relax or even stimulate [23].

During the sessions, the parents were involved in the therapy and decided each time, whether they wanted to relax, to participate in singing or to just observe their child’s reaction to the music. Before the therapy sequence started, the parents were asked about their musical wishes, so their musical and cultural background became part of the therapy and the sessions were shaped by the music therapist and the parents together. As a previous study has shown, parents often started to use their own voice during the therapeutic process, became more confident in this respect and aware how important their singing, humming or even speaking is for their child [24].

After the session and when appropriate, the music therapist provided explanations and feedback to the parents and shared her perceptions of the preterm infant’s reaction to the music. This was done in order to support the parent–infant interaction and to strengthen the parents in their parental role [23].

### 2.4. Data Acquisition

**Figure 1:**
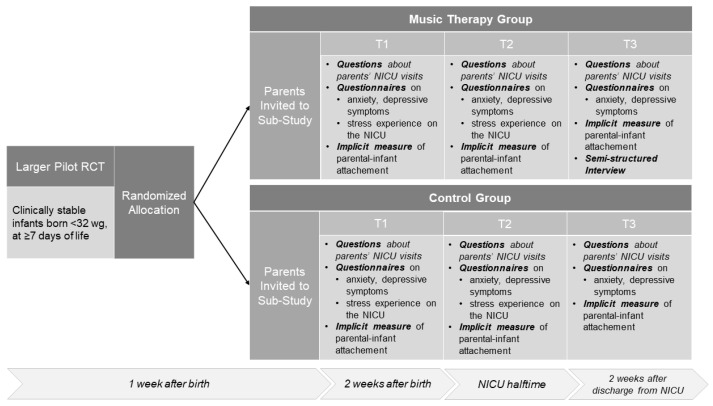
Study design and procedure. Wg = weeks of gestation.

The sub-study was conducted from March 2017 to February 2018 with three assessment time points. The first assessment (T1) was scheduled for two weeks after birth, the second (T2) at approximate halftime of the NICU hospitalization and the third (T3) for two weeks after discharge from the NICU. At each time point (T1, T2, T3), mothers and fathers responded individually and in writing to questions regarding their visits to their infants at the NICU (hours spent at the NICU per day, frequency of kangaroo care, etc.) and completed validated self-report questionnaires measuring parental symptoms of anxiety and depression as well as an implicit measure of parent–infant attachment. Additionally, at T1 and T2, the parents responded to a validated questionnaire on the stress experienced on the NICU. Finally, a semi-structured, problem-centered interview was conducted at T3 with the parents of the MTG and was held separately with mothers and fathers. The design of the study is presented in Figure 1.

One of the first authors (S.M.K.) was responsible for the distribution of the questionnaires. She was blinded to which group the parents had been allocated to. Only shortly before the time of the interviews, which took place at T3, was the first author (S.M.K.) informed which parents were assigned to the MTG.

### 2.5. Psychological Measures

*Symptoms of Anxiety.* Trait anxiety, which refers to a person’s general tendency to experience worries, fears, and anxieties across various situations [35], was measured with the validated German version of the trait subscale of the State-Trait Anxiety Inventory (STAI-t) [36]. Participants are asked to rate 20 items on a four-point Likert scale (1 = *“almost never”*, 4 = *“almost always”*) regarding how they *“generally feel”* (e.g., *“I feel pleasant”*, *“I feel nervous and restless”*). Higher values indicate higher trait anxiety. Cronbach’s alpha ranges between 0.88 and 0.94 [36].

State anxiety, which refers to the degree of anxiety experienced in a current situation, was measured with the validated German short form of the State-Anxiety Inventory (STAI-SKD) [37]. This scale consists of five items. Participants are asked to rate how they feel *“right now”, “at this moment”* (e.g., *“I am tense”*, *“I feel nervous”*) on a four-point Likert scale ranging from 1 = *“not at all”* to 4 = *“very much so”*. Higher scores represent higher state anxiety levels. Cronbach’s alpha for this scale ranges between 0.76 and 0.85 [37].

*Symptoms of Depression.* Depressive symptoms were measured with the well-validated German version of the Edinburgh Postnatal Depression Scale (EPDS) [38,39]. The EPDS is a widely used screening tool for the detection of maternal perinatal depression [40] and has been validated for the assessment of postpartum depressive symptoms in fathers as well [41]. Participants are asked to respond to ten items on a four-point Likert Scale (e.g., from 0 = *“Yes, most of the time”* to 3 = *“No, never”*) regarding how they felt in the past seven days (e.g., *“I have felt sad or miserable”, “I have been so unhappy that I have been crying”*). Cronbach’s alpha for this scale indicates good reliability with a value of 0.81 [39].

*Symptoms of Stress.* Symptoms of stress were measured with the German version of the Parental Stressor Scale: Neonatal Intensive Care Unit (PSS:NICU), which assesses parents’ experience of various stressors caused by the physical and psychosocial environment in the NICU [42,43,44]. Parents are asked to rate a list of 26 potential stressors on a five-point Likert scale ranging from 1 = *“not at all stressful”* to 5 = *“extremely stressful”* based on the degree of stress they were experiencing during the NICU hospitalization of their infant. The PSS:NICU consists of the following three subscales “Sights and Sounds of the Unit” (e.g., “*the sudden noise of the monitor alarm*”), “Infant Behaviour and Appearance” (e.g., “*the small size of my baby”*), and “Parental Role Alteration” (e.g., “*not being able to care for my baby myself”*). Cronbach’s alpha for the sub-scales ranges between 0.73 and 0.92 and for the entire scale between 0.82 and 0.94, indicating good reliability [42,43,44]. The parents responded to the PSS:NICU at T1 and T2, since at T3, the infant already was dismissed from the NICU and at home.

*Attachment.* Feelings of attachment, bonding and connectedness were measured implicitly and non-verbally (i.e., visually) with the Pictorial Representation of Attachment Measure (PRAM) [45]. The mothers and fathers were shown an A4-size paper illustrating a big circle of 18.6 cm diameter in the centre which represented their life. A smaller yellow circle (5 cm diameter) was depicted within the centre of the larger circle and represented the participant him- or her-self. Subsequently, the parents were given a green round sticker (5 cm diameter) labelled “*my baby*” and were asked “*where would you place the baby in your life at the moment?*”. For quantitative analysis, the distance between the centre of the yellow and green sticker was measured (PRAM Self–Baby Distance). A shorter distance indicates a greater feeling of attachment. As an addition to the original PRAM, the parents were asked to place two further green stickers representing (a) the emotional and (b) the physical closeness to their baby. The PRAM has proven to be a valid and easy-to-administer tool to assess parental bonding processes both during the prenatal [45] as well as the postpartum period [46].

### 2.6. Statistical Analysis

Statistical analyses were performed with SPSS (Chicago, IL, USA, version 24) for Windows. During the course of the study, several infants were transferred to another hospital which led to drop-outs in both study groups (cf. Sample Characteristics in the Results) and thus, due to the small sample sizes of both study groups, non-parametric methods were used to analyze the data. Differences between the two groups regarding the psychological measures were explored using the Mann–Whitney test. Potential changes across time (i.e., from T1 to T2 and from T2 to T3) were analyzed with the Wilcoxon signed-rank test. The Bonferroni correction for multiple comparisons was applied where necessary. The level of significance was set at *p* < 0.05 for all analyses (one-tailed for the directional and two-tailed for the non-directional hypotheses).

### 2.7. Qualitative Analysis

The qualitative evaluation of the semi-structured, problem-centered parents’ interview was conducted according to the thematic analysis by Braun and Clarke (2006) [47], which *“involves the searching across a data set—be that a number of interviews or focus groups, or a range of texts—to find repeated patterns of meaning”* [47].

An inductive approach was chosen to identify themes within the data set. In the first step, one of the first authors (S.M.K.) listened to the interviews several times and handwritten notes were made of statements that seemed particularly interesting concerning CMT. In a second step, the interviews were transcribed and by rereading it again, valuable features were marked with color. This was followed by the division into codes, which in turn were organized into sub-themes. The sub-themes were grouped and related to each other until superior themes were found and a definitive presentation could be made. This process resulted as a last step in a final thematic map [47].

## 3. Results

### 3.1. Sample Characteristics

A total of 23 parent couples were asked to participate in this mixed-method study. Due to the need to relocate to other hospitals, a final sample of 16 parent couples with their premature infants was included in this pilot study (*N* = a total of 32 mothers and fathers). Of these 16 couples, ten had been allocated to the MTG (*n =* 20 mothers and fathers) for the main study and six to the CG (*n =* 12 mothers and fathers; Figure S1). By T2, four more infants were transferred to other hospitals leading to 12 parent couples remaining in the pilot study. At this time point, there were eight parent couples (*n* = 16 mothers and fathers) in the MTG and four in the CG (*n* = 8 mothers and fathers). At T3, two further infants were transferred to another hospital. Thus, the study sample was comprised of 10 remaining parent couples of which seven were in the MTG (*n* = 14 mothers and fathers) and three in the CG (*n* = 6 mothers and fathers).

The characteristics of the study population including socio-demographic, pregnancy- and birth-related characteristics are presented in Table 1. There were no significant differences between the MTG and the CG in this regard (*p* > 0.05 for all variables).

At T1, parents in the MTG visited their preterm infants at a median of 3.50 h per day (range 1.50–6.00) and the parents in the CG at a median of 4.00 h per day (range 2.50–6.00). This difference was not significant (*p* > 0.05). In general, mothers stayed longer with their infants (*Mdn* = 4.00 h) than fathers (*Mdn* = 2.50 h), *U* = 43.00, *z* = −2.91, *p* = 0.003, *r* = −0.53. However, there were no significant differences between the mothers in the MTG and the mothers in the CG (*p* > 0.05). All parents (except for one missing value of a mother) had already performed kangaroo care by T1.

At T1, all parents reported speaking very frequently to the infants. There were no significant differences between the two groups in this regard (*p* > 0.05) as well as no significant differences between mothers and fathers (*p* > 0.05). At T1, all parents reported touching their infants very frequently and there were no significant group (*p* > 0.05) or gender differences (*p* > 0.05). The parents expressed the feeling that their infants frequently gave resonance while they were visiting them. There were neither significant group (*p* > 0.05) nor gender differences (*p* > 0.05). Furthermore, the parents indicated that it was very often difficult for them to leave their infants at the NICU in the evenings; there were neither significant group (*p* > 0.05) nor gender differences (*p* > 0.05).

The parents were mostly present during the CMT sessions. A total of 148 (*M* = 13.167) CMT sessions took place, including 48 (*M* = 4.364) individual therapies (i.e., sessions without parents present) and 100 (*M* = 9.091) therapies during kangaroo care. Prior to T1, parents received, on average, two CMT sessions together with their premature infant.

### 3.2. Results of the Quantitative Analyses

#### 3.2.1. Symptoms of Anxiety

The two groups did not differ significantly in trait anxiety levels (STAI-t) at T1, T2, T3 (*p* > 0.05 for all time points). Concerning the course of trait anxiety across time, the reduction from T2 (*Mdn* = 31.50) to T3 (*Mdn* = 29.00) failed to reach the conservative significance level in the MTG at a Bonferroni corrected *p*-value of 0.025 (*z* = −1.85, *p* = 0.034, *r* = −0.349). In the CG, there were no significant changes, neither from T1 to T2, nor from T2 to T3.

**Figure 2:**
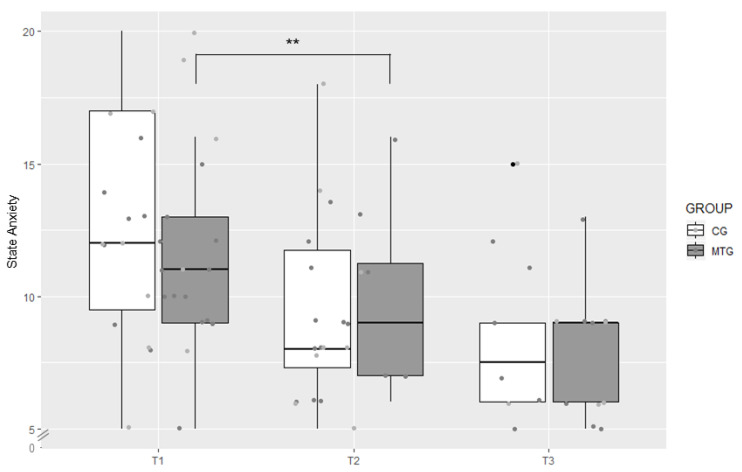
Boxplot of state anxiety levels in the music therapy group (MTG) and the control group (CG) measured at T1, T2, and T3 with the German short form of the state version of the State-Trait Anxiety Inventory, ** *p* < 0.01.

The two groups did not differ significantly in state anxiety levels (STAI-SKD) at T1, T2, T3 (*p* > 0.05 for all time points). However, there was a significant reduction in state anxiety levels from T1 (*Mdn* = 11.00) to T2 (*Mdn* = 9.00), *z* = −2.71, *p* = 0.002, *r* = −0.451, within the MTG, while in the CG, there were no significant changes between T1 to T2 or between T2 to T3 (*p* > 0.05; Figure 2).

#### 3.2.2. Symptoms of Depression

**Figure 3:**
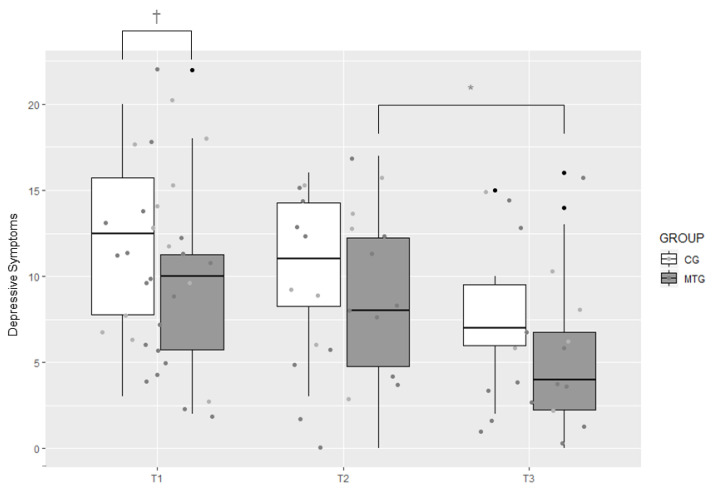
Boxplot of levels of depressive symptoms in the music therapy group (MTG) and the control group (CG) measured at T1, T2, and T3 with the German version of the Edinburgh Postnatal Depression Scale, * *p* < 0.05, ^†^ *p* < 0.10.

At T1, the extent of depressive symptoms (EPDS) in the MTG (*Mdn* = 10.00) was not significantly different from to extent of depressive symptoms in the CG (*Mdn* = 12.50), *z* = −1.44, *p* = 0.077, *r* = −0.255. There were no significant differences between the two groups at T2 and T3 either (*p* > 0.05 for all). However, within the MTG, there was a significant reduction in depressive symptoms from T2 (*Mdn* = 8.00) to T3 (*Mdn* = 4.00) at a Bonferroni corrected *p*-value of 0.025 (*z* = −2.04, *p* = 0.022, *r* = −0.373). There were no significant changes across time in the CG (*p* > 0.06 at a Bonferroni corrected *p*-value of 0.025; Figure 3).

#### 3.2.3. Symptoms of Stress

**Figure 4:**
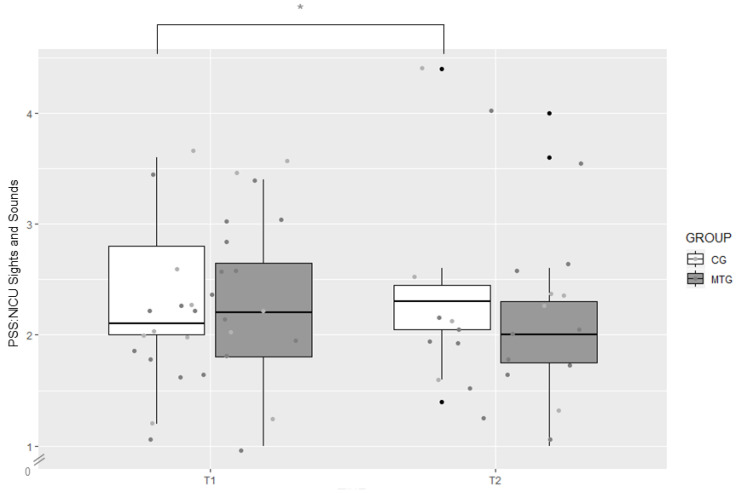
Boxplot of levels of parental stress regarding sights and sounds on the neonatal intensive care unit (NICU) in the music therapy group (MTG) and the control group (CG) measured at T1 and T2 with the German version of the Sights and Sounds subscale of the Parental Stressor Scale: Neonatal Intensive Care Unit (PSS:NICU), * *p* < 0.05.

There were no significant differences between the two groups at T1 and T2 regarding the Sights and Sounds subscale of the PSS:NICU (*p* > 0.05 for both). Within the MTG, there were no significant differences from T1 to T2. However, within the CG, parental stress regarding the Sights and Sounds subscale increased from T1 (*Mdn* = 2.10) to T2 (*Mdn* = 2.30), *z* = −2.39, *p* = 0.016, *r* = −0.534 (Figure 4).

With regard to the Baby Looks and Behaves subscale, there were no significant differences between the two groups at T1 and T2 (*p* > 0.05 for both). There were no significant differences within the MTG and the CG between T1 and T2 (*p* > 0.05 for both).

At T1, the difference between the CG (*Mdn* = 3.07) and MTG (*Mdn* = 2.64) on the Relationship and Parental Role subscale of the PSS:NICU was non-significant, *U* = 72.00, z = −1.87, *p* = 0.062, *r* = −0.331. This was similarly the case at T2 (*p* > 0.05). In relation to the course of parental stress regarding this subscale, no significant results emerged, neither in the MTG nor in the CG (*p* > 0.05 for both).

#### 3.2.4. Attachment

**Figure 5:**
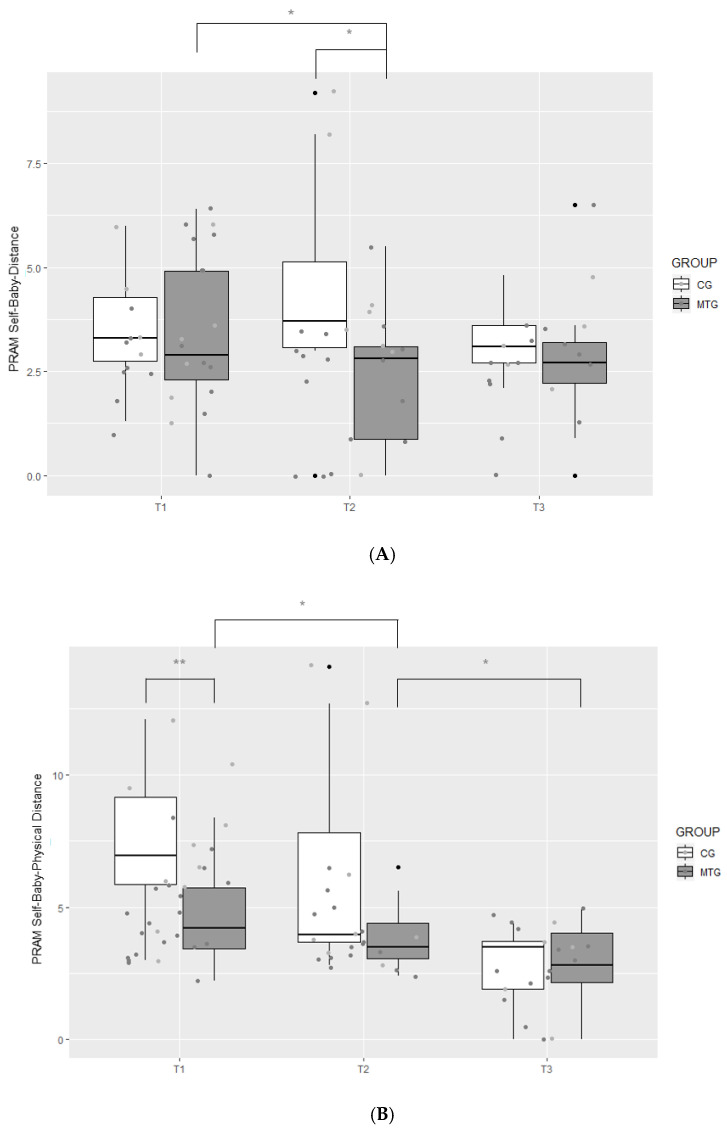
Boxplot of levels of the degree of Self–Baby Distance (**A**) and Self–Baby Physical Distance (**B**) in the music therapy group (MTG) and the control group (CG) measured at T1, T2, and T3 with the Pictorial Representation of Attachment Measure (PRAM), ** *p* < 0.01, * *p* < 0.025.

There was no significant difference between the two groups at T1 regarding the PRAM Self–Baby Distance. However, at T2, the MTG exhibited a significantly shorter distance (*Mdn* = 2.80 cm) compared to the CG (*Mdn* = 3.70 cm), *U* = 28.00, *z* = −2.21, *p* = 0.026, *r* = −0.452. At T3, there were no significant group differences anymore (*p* > 0.05). Within the MTG, the PRAM Self–Baby Distance decreased significantly from T1 at a Bonferroni corrected *p*-value of 0.025 (*Mdn* = 2.90 cm) to T2 (*Mdn* = 2.80), *z* = −2.76, *p* = 0.004, *r* = −0.460 (Figure 5A). There were no differences in the PRAM Self–Baby Distance from T2 to T3 (*p* > 0.05). There were no significant differences within the CG across all time points (*p* > 0.05 for all time points).

Concerning the PRAM Self–Baby Emotional Distance, again there were no significant differences between the two groups at T1 and T3 (*p* > 0.05). Similarly, at T2, the MTG did not exhibit a significantly shorter distance (*Mdn* = 3.10 cm) compared to the CG (*Mdn* = 3.50 cm), *U* = 35.00, *z* = −1.78, *p* = 0.077, *r* = −0.364. There were no significant differences from T1 to T2 and from T2 to T3 within both the MTG and the CG at a Bonferroni corrected *p*-value of 0.025.

At T1, the MTG displayed a significantly shorter distance in the PRAM Self–Baby Physical Distance (*Mdn* = 4.20 cm) compared to the CG (*Mdn* = 6.95 cm), *U* = 39.50, *z* = −2.66, *p* = 0.006, *r* = −0.486. There were no significant group differences at T2 and T3 (*p* > 0.05). However, in the MTG, there was a significant decrease in distance from T1 (*Mdn* = 4.20 cm) to T2 (*Mdn* = 3.50 cm), *z* = −2.36, *p* = 0.016, *r* = −0.399, and again from T2 to T3 (*Mdn* = 2.80 cm), *z* = −2.34, *p* = 0.016, *r* = −0.435, at a Bonferroni corrected *p*-value of 0.025 (Figure 5B). No such changes across time were apparent in the CG.

### 3.3. Results of the Qualitative Analysis

**Figure 6:**
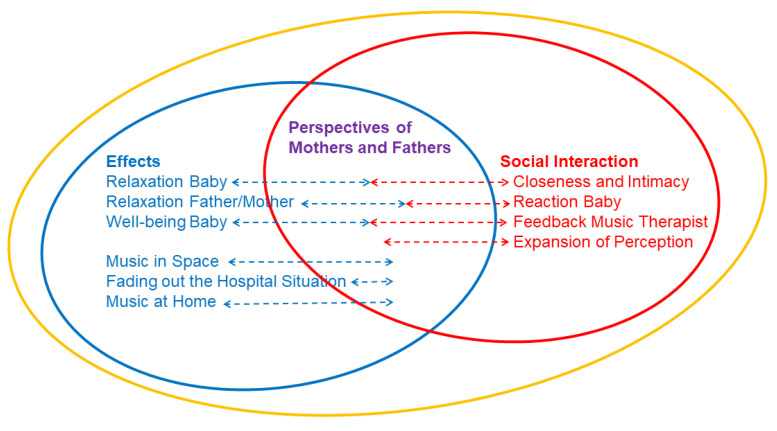
Graphic representation of the three main and six sub-themes that emerged from the thematic analysis.

Three main themes emerged from the thematic analysis: Effects, Perspectives of Mothers and Fathers, and Social Interaction. The three topics are interrelated and mutually dependent, as can be seen in the graphic representation (Figure 6). For example, parents and their infant could often develop more intimacy when their infant seemed relaxed, or vice versa, because the parents were relaxed, they were much more receptive to their infants’ reactions, so that they communicated and related more intensely with their infant.

In general, it was remarkable how all parents in the interviews were, without exception, very quick to talk about CMT’s effects and this without having been asked directly about it. As one of the three main themes, Effects are composed of six sub-themes: Relaxation Baby, Relaxation Father/Mother, Well-being Baby, Music in Space, Fading out the Hospital Situation and Music at Home.

The effect of Relaxation was a predominant topic in the interviews. Sometimes it referred to the parents themselves, who described how they could relax due to CMT, sometimes to the premature infant and its well-being or to the spatial environment, which was influenced by the music and thus created a more peaceful atmosphere.

“*I have the feeling that he could let himself go. He was so relaxed on top of me and had a satisfied expression to me* […].”

(M49)

“*He had quite a lot of* [oxygen] *decreases at the beginning and with the music, he really never had any decreases when I was there. That was always reassuring for me, just that it was good for him—I really have the feeling that it was good for him*.”

(M55)

Concerning the environmental influence, the parents mentioned that the music facilitated a protective and enveloping space for them and their infants and allowed them to fade out the alarms’ beeping and the restless hospital atmosphere for a moment.

“*That was the moment when you just could relax. I could never do that usually* […] *Just us—for me, it was really our moment*.”

(M72)

“*You could relax; you also forgot that you are not alone here with S.* [daughter] […] *You didn’t hear or see the monitors anymore, she* [the music therapist] *was always watching*.”

(M72)

“*At the IMC* [Intermediate Care], *you really noticed when music therapy took place. Everyone was relaxed, all caregivers* […] *It immediately became quiet on the unit. It was radiant*.”

(M72)

Furthermore, most parents reported that music continued to accompany them in their everyday life. Through the experience with CMT, they realized the effect of their vocal attention on their premature infant and could relax in the face of their high demands regarding singing.

“*Now I sing with a feeling that it will surely do him good. I don’t know if I would have perceived it that way before*.”

(M49)

“*I take home with me that we increasingly sing and hum with him* […] *and not that you have to be a professional musician or a professional singer to sing something to him* […] *he doesn’t care if you sing out of tune. The most important thing is music*.”

(M48)

Social Interaction as the second overarching theme consists of the following sub-themes: Closeness and Intimacy, Reaction Baby, Feedback Music Therapist, Expansion of Perception.

The parents reported that they experienced closeness and intimacy during CMT. The music, humming and the monochord’s vibrations, which were passed on from the parents to their premature infant, seemed to create a new way of connecting.

“*Yes, you feel more as one, because of the monochord. I can feel the vibrations that go through me to him and the humming in addition—I felt I was more united*.”

(M48)

Additionally, the support of and relationship to the music therapist enriched these feelings. This circumstance was often mentioned in the parents’ interviews and appreciated. The parents stated that the music therapist’s feedback facilitated a new and different view of their child and helped them even to get to know him/her better.

“*For me, it simply wasn’t so natural in our situation and I was then* [during CMT] *allowed to simply observe L*. [son]—*and even a little—get to know him better and also get to know him differently during this time of music therapy.*”

(M62)

Furthermore, the infant’s positive reactions during CMT revealed to the parents that their child is capable of responding to the environment, and in many cases, this reaction triggered a sequence of relationship replies, so that communication could develop. This circumstance often calmed and relaxed the parents and gave them a feeling of security.

“*That* [CMT*] was always a real highlight. Not only for L.* [son]. *The music therapist said, he always reacted very well. She explained to me what she saw and then I could observe it* […] *It also always reassured me and it was good for me, very good*.”

(M62)

The third main theme Perspectives of Mothers and Fathers, arose from the motivation to include fathers and their perspectives in the research topic in order to identify possible differences in mothers’ and fathers’ perspectives, behaviors or coping strategies.

The interviews revealed small differences in the details of the description of CMT: if fathers tended to be more objective in their choice of words, mothers described the effects of CMT in more detail and with more adjectives and superlatives. Fathers also often talked about the continuation of music in everyday life. The purchase and use of an instrument as an additional way of making music at home was mostly mentioned by fathers and seemed to have created much joy and interest.

“*I enjoy it, also the instrument* [bought an instrument for home use after the experience with CMT]. *It is a beautiful wooden instrument. I find it a good thing*.”

(V48)

Generally, through CMT, the fathers experienced that they have just as much vocabulary as the mothers in the musical communication with their premature infant. This is all the more important because they naturally often play a secondary role in this phase shortly after birth. CMT encouraged them in terms of their importance as fathers. Later in everyday life, they mentioned that music often became a ritual of father–child communication and/or was used by them to soothe their child.

“*Yes, we sing and hum a lot with her* [daughter] […] *As soon as she is with her daddy in the evening and he begins to hum, she is just quiet. She needs that, she is used to it*.”

(M72)

None of the parents expressed any negative experience with the music therapy.

## 4. Discussion

Premature birth is a stressful life event for the infant and the parents [10,11,12,13,14], and both are at risk of increased stress, which can affect the parent–infant dyad [16,17,18,19]. Hence, we aimed to evaluate whether CMT can alleviate anxiety, stress and depressive symptoms in the parents and support the bonding process between the parents and their infant.

Our first hypothesis that parents of premature infants treated with CMT show less anxiety and depressive symptoms than the CG parents can partially be confirmed by the present study. Our quantitative analysis showed no significant overall difference in anxiety and depressive symptoms between the MTG and the CG. However, a significant reduction in state anxiety levels from T1 to T2 was evident within the MTG, and there was also a trend towards a significant reduction in trait anxiety levels within the MTG from T2 to T3. No such changes were apparent in the CG.

Interestingly, we could already show a significant reduction in state anxiety levels in the MTG from T1 to T2, after the parents had experienced, on average, six therapy sequences. From this, it can be assumed that CMT could rapidly trigger a positive effect on the parents. The trend towards a significant reduction in trait anxiety within the MTG from T2 to T3 suggests that CMT may have a lasting effect. Although the MTG and CG converge on their anxiety level at T3, our results indicate that parents with CMT can be supported during their hospital stay by more rapidly reducing their initial anxiety levels.

Concerning the STAI, previous studies have reported a significant dampening effect of live music therapy on maternal anxiety [31,32,33]. Considering the fact that all these studies had a higher number of participants, our results need to be reviewed with a larger sample size.

We also found that at T1, there was a trend towards significantly lower depressive symptoms (EPDS) in the MTG compared to the CG and there was a significant reduction in depressive symptoms within the MTG from T2 to T3. No such changes were apparent in the CG.

Given the fact that depressive and anxiety symptoms tend to be related [48,49], a persistent anxiety state could lead to depressive symptoms. The significant reduction in state anxiety levels from T1 to T2 within the MTG and the later trend towards a significant reduction in trait anxiety could be in relation to the just mentioned results regarding the significant reduction in depressive symptoms within the MTG between T2 and T3 and the trend towards significantly lower depressive symptoms in the MTG compared to the CG at T1. CMT may offer a possibility to alleviate the parents’ fears of loss, failure and anxiety early enough to reduce or prevent the development of depressive symptoms. Empowering parents of premature infants through musical engagement, as reported in the qualitative case study by Haslbeck [25], could play an important role concerning these outcomes. By offering parents the opportunity to express themselves towards their premature infant responsively and joyfully, and by supporting their autonomy by being creative in their own language and cultural heritage, empowerment can emerge, and the passive emotions of fear and anxiety can be transformed into more active and appealing feelings [25].

Therefore, CMT performed at the NICU with the parents and their premature infants can also be seen as a coping strategy in the stress situation itself. This view refers to the coping strategy “Applied Relaxation” by Lars Göran-Öst developed in the 1970s, which was first used in the treatment of phobias and panic attacks, later also in generalized anxiety disorders, and is still considered as an effective form of therapy [50]. This technique assumes the best possible effectiveness against anxiety and stress if anxiety signals are recognized as early as possible and counteracted or even averted by applying the relaxation techniques in the stress situation itself [50].

A study by Ionio et al. [13] found that both mother and father are at a greater risk of developing higher levels of anxiety, depression, anger, and stress after experiencing the premature birth of their child. Therefore, they call for family-centered interventions from the first moment onwards to support the parents and allow them to actively participate in the development of their infant [13]. So does the study by Forcada-Guex et al. [17], which demonstrated that premature birth affects mother–infant interactions and, in particular, that high maternal posttraumatic stress symptoms were related to a “controlling” mother–infant interaction pattern [17]. In their conclusion, they emphasize the need to identify parents who have experienced a trauma related to a premature birth and underline the importance of providing them the opportunity of joint observations and care for their infant in order to promote parental feelings and competences [17].

The assumption that CMT can have a stress-relieving effect is consistent with the results of our qualitative analyses, reflected by the sub-theme Relaxation Father/Mother, Relaxation Baby. This topic is also mentioned in the studies by Ettenberger et al. [31] and Haslbeck [25] where music therapy showed a valuable effect on the parents’ well-being. Ettenberger et al. [31] point out that the ability to relax and participate in a joyful musical activity is crucial in helping parents to cope with the stress they experience in the NICU and may contribute to creating a more optimal parent–infant relationship [31].

This leads us to our second hypothesis, that the MTG parents and the CG parents differ in their stress and attachment levels towards their premature infant. If the course has already been discussed concerning the stress symptoms of parents of premature infants, the following applies to the course of the bonding behavior.

This hypothesis can partially be confirmed. At T1, the MTG displayed a significantly shorter distance in the PRAM Self–Baby Physical Distance compared to the CG, and within the MTG, there was a significant decrease in the distance over time. No such changes across time were apparent in the CG. Concerning the PRAM Self–Baby and the PRAM Self–Baby Emotional Distance, trends toward significant changes were observed between T1 and T2, indicating closer distances for the MTG.

Regarding these results, we take Ettenberger et al.’s assumption [31] even further and suggest that CMT, through relaxation, invites the parents in an easy manner to care for the relationship with their premature infant so that dialog and closeness can take place more often. This observation is reflected in one of the three main themes (Social Interaction) in our qualitative analysis. The music, the humming, and possibly the monochord‘s vibration during the therapy session which was passed on from the parents to their premature infants, seemed to create an additional sense of closeness and intimacy, which subsequently enabled a new way of meeting and being together [25].

The instrument monochord could play a valuable and promising role concerning this result. It makes the music not only audible but also feelable for both the parents and their premature infant. This may explain, among other things, the feeling of unity with their infant described by many parents during the qualitative interviews, reflected within the sub-theme Closeness and Intimacy. However, further research on this assumption is necessary and would certainly be interesting.

In their review on the impact of suboptimal bonding caused by prematurity, Kommers et al. [15] conclude that negative effects could be reversed by bonding interventions and that NICUs should focus more on this aspect [15]. From their research on maternal postpartum behavior and its implication for the mother–infant and father–infant relationship, Feldman and Eidelman [16] conclude that following premature birth, mothers of premature infants were less capable of coordinating their social behavior with the few moments of alertness of the infant [16]. Therefore, they advocate supporting techniques for parents of premature infants and propose methods linked to mother–infant touch and contact as well as psychosocial interventions.

We are convinced that CMT can be an appropriate therapy approach in this field, as it combines corporeality and unconscious parts of therapy with a psychosocial direction. CMT may support premature infants in developing their communicative abilities and may empower the parents by allowing them to experience inter-subjectivity with their infant through music [25]. Therefore, we call CMT not a parental education method, but rather a therapeutic method to use with the parents and to elicit their endogenous parental abilities. Often, not many words are needed, as music with its resonance does its job entirely, by opening up a space where parents and infants get into the same pulsation and experience intimate and intersubjective moments [25]. This is reflected in our qualitative results through the sub-theme Expansion of Perception and is closely related to the sub-theme Feedback Music Therapist, since the music therapist’s sensitive behavior and musical response to the needs of the parents and their infants contribute substantially to the possibilities of interactions.

At this point, we refer to Benzies et al. [22], who, in their paper examining key components of early intervention programs for preterm infants and their parents, report that studies with positive outcomes for the parents were often associated with improved outcomes for the premature infants. However, they underline that parental education alone does not reduce stress. Apparently, it requires more than education [22].

Nevertheless, it is not yet clear, which factors play an influential role in this form of therapy, and more work is needed to define them better. Additionally, whether the positive results concerning the MTG are due to the music itself, the parents’ participation and empowerment, or related to the fact that the parents received more support and attention in the MTG, should be investigated in future studies. Presumably, all factors together may contribute to the beneficial outcomes of CMT.

Due to the small sample size, it is not easy to draw clear conclusions. This is of course the main limiting factor of the present study. Besides, a larger sample size would have enabled us to address further important research questions concerning music therapy on the NICU, such as the influence of the number of therapy sessions on parental well-being and attachment. Moreover, we would have been able to evaluate the data of the mothers and fathers separately in the quantitative analyses and thus, gain a better understanding of the respective needs of mothers and fathers. A third limiting factor concerns the fact that CMT sessions had already begun prior to the T1 questionnaire assessment. Thus, true baseline data regarding parental symptoms of depression, anxiety and stress as well as the parent–infant attachment is lacking.

One strength of this study is its mixed-method design, since it provides a more in-depth understanding of the parents’ psychological processes and their point of view. A further strength concerns the inclusion of both mothers and fathers as well the use of an implicit measure of parent–infant attachment (i.e., the PRAM) combined with the explicit stress questionnaires. The use of these various methods should be expanded in future studies on the effect of CMT for parents in the NICU.

## 5. Conclusions

Overall, this study’s results support the assumption that CMT may have the potential to alleviate symptoms of anxiety, depression and stress in premature infants’ parents during their hospitalization and may thereby support the parent–infant bonding process right from the very beginning. The results call for a more extensive powered follow-up study to further investigate the potential of CMT on parental well-being and parent–infant bonding.

## Figures and Tables

**Table 1:** Characteristics of the study population (*N* = 32).

	Music Therapy Group	Control Group
Participants (*n*)	20	12
Couples (*n*)	10	6
Maternal age (years) (median (range))	31.50 (26.00–43.00)	30.00 (27.00–35.00)
Marital status (% (*n*))		
Married	50 (*n* = 10)	58 (*n* = 7)
Unmarried	50 (*n* = 10)	42 (*n* = 5)
Cohabitating (% (*n*))	100 (*n* = 20)	100 (*n* = 12)
Maternal nationality (% (*n*))		
Swiss	90 (*n* = 9)	50 (*n* = 3)
German	10 (*n* = 1)	17 (*n* = 1)
Jordanian		17 (*n* = 1)
Croatian		17 (*n* = 1)
Maternal language (% (*n*))		
German	100 (*n* = 10)	83 (*n* = 5)
Arabic		17 (*n* = 1)
Maternal educational qualification (% (*n*))		
Apprenticeship	40 (*n* = 4)	50 (*n* = 3)
University of applied sciences degree	30 (*n* = 3)	
University degree	30 (*n* = 3)	50 (*n* = 3)
Paternal nationality (% (*n*))		
Swiss	70 (*n* = 7)	67 (*n* = 4)
German	20 (*n* = 2)	33 (*n* = 2)
Kosovar	10 (*n* = 1)	
Paternal language (% (*n*))		
German	90 (*n* = 9)	100 (*n* = 6)
Albanian	10 (*n* = 1)	
Paternal educational qualification (% (*n*))		
Apprenticeship	60 (*n* = 6)	50 (*n* = 3)
University of applied sciences degree	10 (*n* = 1)	17 (*n* = 1)
University degree	30 (*n* = 3)	33 (*n* = 2)
Primigravida (% (*n*))	80 (*n* = 8)	67 (*n* = 4)
Primiparous (% (*n*))	80 (*n* = 8)	67 (*n* = 4)
Sectio Caesarea (% (*n*))	100 (*n* = 10)	100 (*n* = 6)
Total number of infants	11	8
Set of twins (% (*n*))	10 (*n* = 1)	33 (*n* = 2)
Male infants (% (*n*))	82 (*n* = 9)	50 (*n* = 4)
Gestational age at birth (weeks) (median (range))	28.43 (24.86–31.43)	28.29 (25.00–29.00)
Birth weight (g) (median (range))	1070 (680–1590)	785.00 (600–1310)
Birth size (cm) (median (range))	38.00 (33.00–43.00)	34.50 (32.00–39.00)
Apgar score (10 min) (median (range))	10 (7–10)	9 (6–10)

Note: The music therapy and control group showed now significant differences in socio-demographic, pregnancy- and birth-related characteristics (*p* > 0.05 for all variables).

## Data Availability

The data presented in this study are available on request from the corresponding author. The data are not publicly available due to privacy and ethical reasons.

## Supplementary Materials

The following are available online at https://www.mdpi.com/1660-4601/18/1/265/s1, Figure S1: Study flow chart. At T1, there were 20 mothers and fathers in the Music Therapy Group (MTG) and 12 in the Control Group (CG). At T2, 16 mothers and fathers remained in the MTG and 8 in the CG. Finally, at T3, 14 mothers and fathers remained in the MTG and 6 in the CG.
