# Supplementary material for: Creative Music Therapy with Premature Infants and Their Parents: A Mixed-Method Pilot Study on Parents’ Anxiety, Stress and Depressive Symptoms and Parent–Infant Attachment
Source: Int J Environ Res Public Health. 2020 Dec 31;18(1):265. doi: 10.3390/ijerph18010265 (PMC7795112; doi:10.3390/ijerph18010265)
Supplement: Supplementary file 1 [file ijerph-18-00265-s001.pdf]

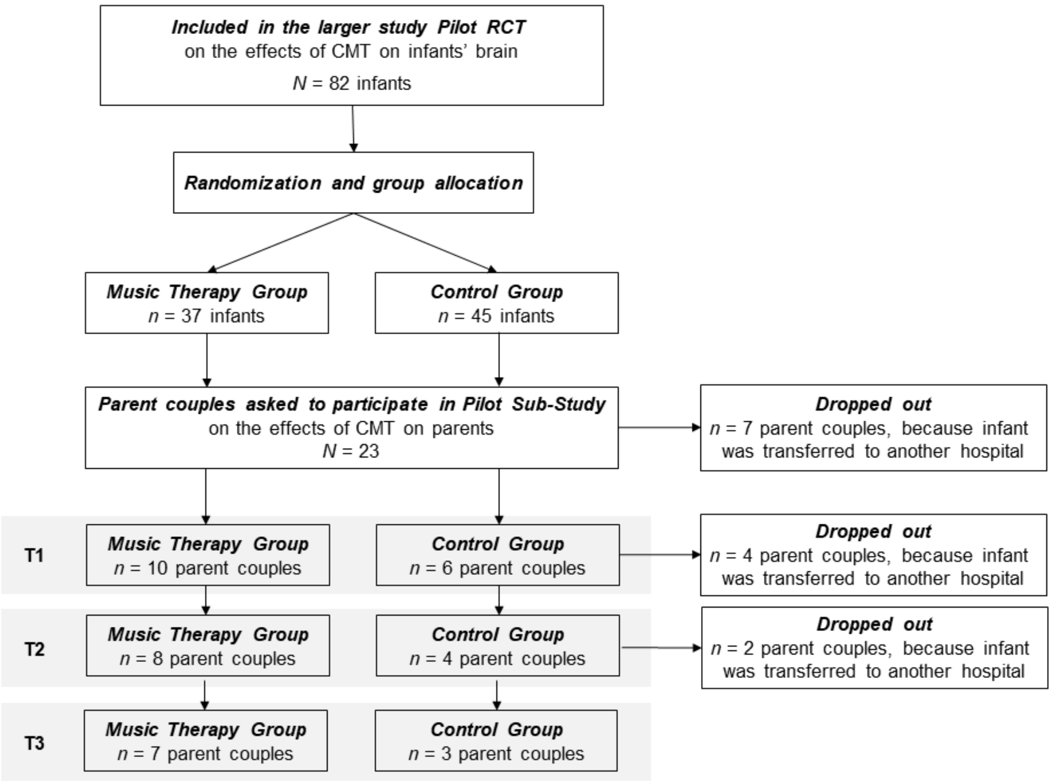

Figure S1. Study flow chart. At T1, there were 20 mothers and fathers in the Music Therapy Group (MTG) and 12 in the Control Group (CG). At T2, 16 mothers and fathers remained in the MTG and 8 in the CG. Finally, at T3, 14 mothers and fathers remained in the MTG and 6 in the CG.
